# Beyond Words: A Case of Pure Alexia Following Posterior Cerebral Artery Occlusion

**DOI:** 10.7759/cureus.52734

**Published:** 2024-01-22

**Authors:** João Romano, Sara Silva, Nuno Oliveira, Fausto Carvalheira, João Paulo Sousa

**Affiliations:** 1 Ophthalmology, Centro Hospitalar de Leiria, Leiria, PRT

**Keywords:** hemianopsia, homonymous quadrantanopia, ischemic stroke, posterior cerebral artery, pure alexia

## Abstract

Alexia is an acquired reading disorder known as pure alexia or alexia without agraphia when unaccompanied by other higher-level deficits. We present the case of a 40-year-old man experiencing a sudden-onset headache and blurred vision. Despite an absence of known medical history, the patient exhibited a distinctive difficulty in reading without impairing other language aspects accompanied by a right superior homonymous quadrantanopia. Through comprehensive ophthalmological and neurological evaluations, a diagnosis of pure alexia was established. An imaging scan uncovered a left posterior cerebral artery occlusion as the underlying cause. Meticulous assessments of visual acuity, perimetry, and non-visual functions played a pivotal role in decisively diagnosing this condition. This case emphasizes the indispensable role of ophthalmologists in recognizing urgent clinical conditions that extend beyond ophthalmic concerns.

## Introduction

Alexia was one of the first disconnection syndromes to be described and is an acquired reading disorder [1,2]. It can manifest as an inability to read or read more slowly than before, after excluding sensory deficits, aphasic symptoms, or cognitive deficits [1,2]. According to the literature, deficits at a higher level have received less investigation, but it is known that they relate to the recognition of more complex stimuli, such as faces (prosopagnosia), familiar places (topographagnosia), or words (alexia) [3].

In the early stages, alexia is often accompanied by anomic aphasia, homonymous hemianopia, and memory deficits [1,4]. It can be accompanied by agraphia when there is a compromise of speech and language [1,2,4]. There are cases of pure alexia or alexia without agraphia, in which the patient only presents with alexia, although superficial dysgraphia may exist [1,4].

The causes of alexia are diverse: ischemic stroke (more frequent), tumors, Alzheimer's disease, and trauma [1,3-5]. Pure alexia is more commonly associated with stroke [1]. The posterior cerebral artery supplies the cortex of the occipital and ventral temporal lobes, which are related to the recognition of lower-level visual stimuli (such as hemianopia) or higher-level (including alexia) [3-5].

Here, we report a case of pure alexia, in which there was difficulty in reading without compromising other aspects of language. We describe its diagnosis and the collaboration between ophthalmology and neurology.

## Case presentation

**Figure 1:**
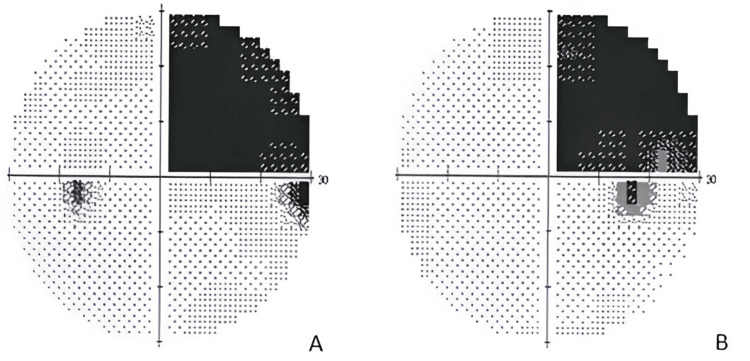
Standard 30-2 computerized perimetry of the left (A) and right (B) eyes showing a right superior homonymous quadrantanopia (“pie in the sky”).

A 40-year-old Caucasian male with no known medical history or regular medications presented to the emergency department with a sudden headache and blurred vision for one day. During the patient's evaluation, he was cooperative and attentive to questions, with no impairment of non-visual language. The patient was unable to discern whether the vision reduction was more pronounced in the right or left eye. A general ophthalmological evaluation revealed a corrected distance visual acuity of 20/20 in each eye. Pupillary photomotor reflexes were normal and ocular motility was entirely intact. Anterior segment evaluation and fundoscopic examination revealed no obvious abnormalities. During near visual acuity assessment, the patient could identify individual letters but was unable to read words. Formal perimetry (Figure 1) presented a right superior homonymous quadrantanopia (“pie in the sky”). Evaluation of faces and objects was conducted without detectable flaws.

**Figure 2:**
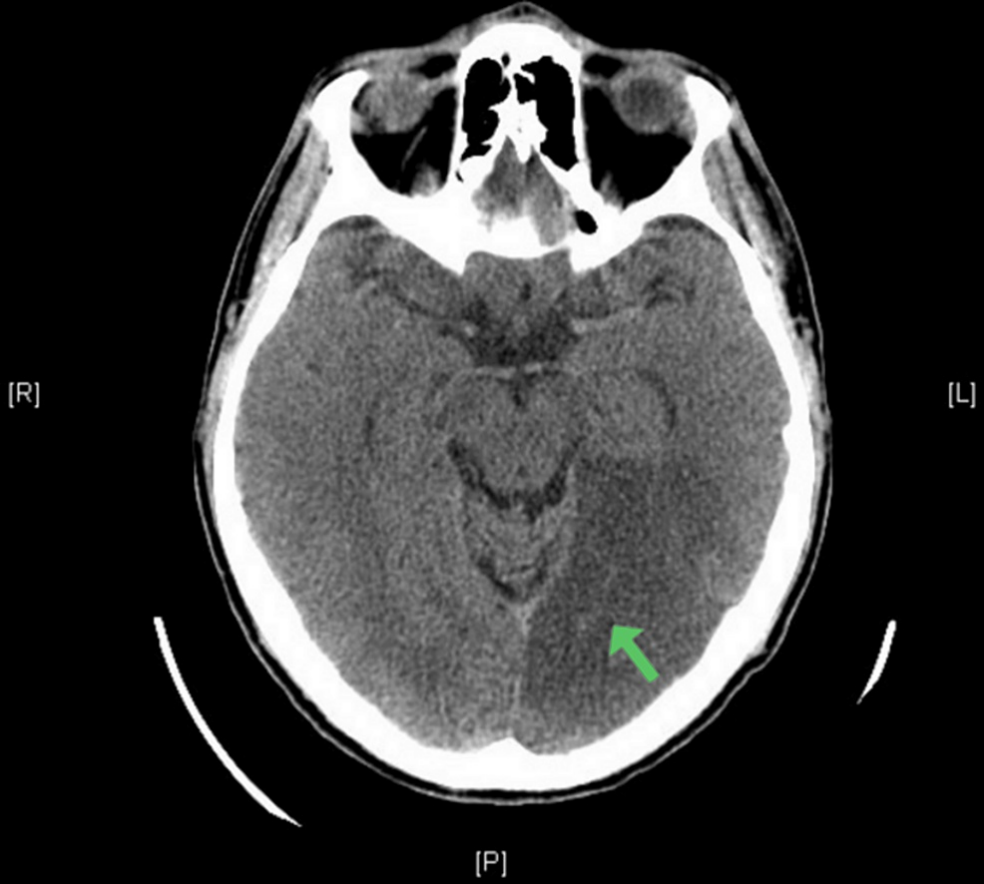
Hypodense occipitotemporal cortical-subcortical lesion on the left side (green arrow) in the contrast-enhanced computed tomography of the brain.

He was referred to the neurology department due to suspected reading impairment (alexia) and homonymous visual field defect. Subsequent contrast-enhanced computed tomography of the brain revealed a hypodense occipitotemporal cortical-subcortical lesion on the left side, leading to sulcal effacement (Figure 2). This radiological finding was suggestive of a recent ischemic event in the territory of the ipsilateral posterior cerebral artery. The definitive diagnosis of ischemic stroke was established, requiring hospitalization. After three days of hospitalization, the patient was discharged home on dual antiplatelet therapy, still presenting mild alexia.

## Discussion

In the presented case, the cause of alexia was the occlusion of the posterior cerebral artery in the dominant cerebral hemisphere (left). This is in line with the literature and recent studies have suggested that the dominant hemisphere plays a predominant role in word recognition, although the right hemisphere can also contribute [1,3-5]. While prosopagnosia and topographagnosia are associated with right or bilateral occipitotemporal lesions, in alexia, the typical site of involvement is the left ventral occipitotemporal cortex, also known as the "visual word form area" [1]. Additionally, the localized damage in this case manifests exclusively as visual disturbances, with no accompanying sensory or motor deficits.

Careful perimetry and visual acuity assessment to exclude other possibilities that could account for the reading impairment is important. Non-visual functions also need to be thoroughly tested in the clinical case, as the patient's cooperation during visual tests requires preserved oral and auditory language. In the current case, the communication pathway between the auditory centers and language centers remained intact, enabling the ability to interpret words spelled out to or by the individual.

The cornerstone aspect in this case was the assessment of near visual acuity. Unlike distance visual acuity tests that involve naming individual letters, evaluating near visual acuity requires the comprehension of written language. This distinction played a crucial role in uncovering the patient's specific reading impairment. This atypical reading pattern suggested a deficiency, alexia, in favor of a specific cerebral etiology rather than an ocular pathology. Alexia is often linked with a contralesional visual field defect [1], which may be hemianopia or quadrantanopia depending on the location of the lesion [6]. In this case, the ischemic stroke affected the Meyer’s loop in the temporal lobe resulting in a right superior homonymous quadrantanopia, colloquially referred to as "pie in the sky” [7]. CT scan confirmed the stroke, but thrombolytic therapy was not administered to the patient. This decision was in line with current guidelines, as the patient presented more than 24 hours from their last known well time, surpassing the recommended therapeutic window [8].

At the time of discharge, the patient had mild pure alexia, which persisted. It is expected that such patients will partially recover their reading ability, but most of them remain with mild pure alexia [1,3]. For recovery, auditory communication and audiobooks may play a crucial role [3].

## Conclusions

This case underscores the critical role of ophthalmologists in identifying urgent clinical conditions, even beyond usual ophthalmic concerns. The visual symptoms, specifically the presented reading disorder (pure alexia), served as a crucial indicator, enabling the timely recognition of an ischemic stroke with left posterior cerebral artery occlusion and ensuring appropriate referral. The collaborative approach between ophthalmology and neurology played a pivotal role in distinguishing such intricate neurological conditions as pure alexia.
